# Antigen-sensitized CD4^+^CD62L^low ^memory/effector T helper 2 cells can induce airway hyperresponsiveness in an antigen free setting

**DOI:** 10.1186/1465-9921-6-46

**Published:** 2005-05-28

**Authors:** Kazuyuki Nakagome, Makoto Dohi, Katsuhide Okunishi, Yasuo To, Atsushi Sato, Yoshinori Komagata, Katsuya Nagatani, Ryoichi Tanaka, Kazuhiko Yamamoto

**Affiliations:** 1 Department of Allergy and Rheumatology, Graduate School of Medicine, University of Tokyo, Tokyo, Japan

## Abstract

**Background:**

Airway hyperresponsiveness (AHR) is one of the most prominent features of asthma, however, precise mechanisms for its induction have not been fully elucidated. We previously reported that systemic antigen sensitization alone directly induces AHR before development of eosinophilic airway inflammation in a mouse model of allergic airway inflammation, which suggests a critical role of antigen-specific systemic immune response itself in the induction of AHR. In the present study, we examined this possibility by cell transfer experiment, and then analyzed which cell source was essential for this process.

**Methods:**

BALB/c mice were immunized with ovalbumin (OVA) twice. Spleen cells were obtained from the mice and were transferred in naive mice. Four days later, AHR was assessed. We carried out bronchoalveolar lavage (BAL) to analyze inflammation and cytokine production in the lung. Fluorescence and immunohistochemical studies were performed to identify T cells recruiting and proliferating in the lung or in the gut of the recipient. To determine the essential phenotype, spleen cells were column purified by antibody-coated microbeads with negative or positive selection, and transferred. Then, AHR was assessed.

**Results:**

Transfer of spleen cells obtained from OVA-sensitized mice induced a moderate, but significant, AHR without airway antigen challenge in naive mice without airway eosinophilia. Immunization with T helper (Th) 1 elicited antigen (OVA with complete Freund's adjuvant) did not induce the AHR. Transferred cells distributed among organs, and the cells proliferated in an antigen free setting for at least three days in the lung. This transfer-induced AHR persisted for one week. Interleukin-4 and 5 in the BAL fluid increased in the transferred mice. Immunoglobulin E was not involved in this transfer-induced AHR. Transfer of *in vitro *polarized CD4^+ ^Th2 cells, but not Th1 cells, induced AHR. We finally clarified that CD4^+^CD62L^low ^memory/effector T cells recruited in the lung and proliferated, thus induced AHR.

**Conclusion:**

These results suggest that antigen-sensitized memory/effector Th2 cells themselves play an important role for induction of basal AHR in an antigen free, eosinophil-independent setting. Therefore, regulation of CD4^+ ^T cell-mediated immune response itself could be a critical therapeutic target for allergic asthma.

## Background

Bronchial asthma is a chronic disorder characterized as reversible airway obstruction, eosinophilic airway inflammation, mucus hypersecretion, and airway hyperresponsiveness (AHR) [1]. In the process of airway inflammation, various types of cells, such as eosinophils, mast cells, T lymphocytes, and dendritic cells are involved [2,3]. AHR to nonspecific stimuli is a hallmark of asthma. However, the precise mechanism to induce AHR has not been fully elucidated. Persistence of eosinophilic airway inflammation is closely linked to induction of AHR [1,4,5]. However, the dissociation of AHR and eosinophilic airway inflammation often occurs [6-12]. For example, Leckie *et al*. showed that administration of neutralizing antibody (Ab) to interleukin (IL) -5 does not suppress AHR despite this treatment abrogating eosinophilia in blood and sputum [6]. In addition, even a protective role of eosinophils on the AHR induction has been recently proposed in a mouse model [9]. These findings suggest that other mechanism(s) than eosinophilic inflammation would be involved in inducing AHR.

On the other hand, in patients with asthma, activated T cells, especially CD4^+ ^T helper (Th) 2 cells, also infiltrate into the airway, which is associated with disease severity [13-15]. In a mouse model, administration of blocking Ab to CD4 suppresses AHR and airway inflammation [11,16]. Moreover, transfer of CD4^+ ^Th2 cells into naive mice and subsequent antigen-inhalation induce AHR and airway inflammation [17,18]. These findings suggest that T cells, especially CD4^+ ^Th2 cells, are also important for the AHR induction. However, in these studies, AHR induced by CD4^+ ^Th2 cells accompanies eosinophilic airway inflammation. Therefore, it remains unclear whether T cells alone could directly induce AHR.

We previously reported that systemic antigen sensitization alone directly induces AHR before development of eosinophilic inflammation in mice [19]. This raised a possibility that systemic immune response to antigen itself could directly induce AHR.

The purpose of the present study was to investigate which component in the immunocompetent cells could directly induce AHR. In this study, we found that passive cell transfer of spleen cells obtained after antigen-sensitization reconstituted AHR in naive mice. Then, using this system, we studied the cell source essential for the AHR induction, and confirmed that antigen-sensitized CD4^+^CD62L^low ^memory/effector Th2 cells would play an essential role for induction of basal AHR.

## Methods

### Immunization of mice and transfer of spleen cells

Mice were immunized as reported previously [19,20]. Seven-week-old male BALB/cAnNCrj mice (Charles River Japan, Kanagawa, Japan) or IL-4 gene-deleted mice (BALB/c-*IL4*^*tm2Nnt*^; Jackson Laboratory, Bar Harbor, ME) were sensitized with an i.p. injection of 2 μg ovalbumin (OVA; Sigma, St. Louis, MO), bovine serum albumin (BSA; Wako, Osaka, Japan), or keyhole limpet hemocyanin (KLH; Calbiochem, LaJolla, CA) plus 2 mg aluminum hydroxide (alum) on days 0 and 11. Control mice received an injection of physiologic saline (SA) without alum on days 0 and 11. Some control mice received an injection of SA plus alum. In some experiments, we used complete Freund's adjuvant (CFA; Difco Laboratories, Detroit, MI) instead of alum as an adjuvant. On day 18, cell suspensions of spleens were obtained by pressing the tissues through a 70-μm nylon filters. Spleen cells prepared from the OVA-sensitized mice (1 × 10^6^, 5 × 10^6^, 2 × 10^7^, or 5 × 10^7 ^in 0.5 ml HBSS, respectively) or the SA-treated mice (5 × 10^7^) were transferred into syngenic recipients by intravenous injection. In some experiments, mice were sensitized with OVA on days 0 and 11, and then challenged with 3% OVA for 10 minutes from day 18 to day 20. On day 21, lungs were excised to observe eosinophilic airway inflammation. All animal experiments were approved by the Animal Research Ethics Board of the Department of Allergy and Rheumatology, University of Tokyo.

### Measurement of airway responsiveness (AR)

On day 22 (4 days after the transfer), AR to methacholine (Mch) was measured with the enhanced pause (Penh) system (Buxco, Troy, NY) as described previously [19,20]. In some experiments, AR was assessed by measurement of airway resistance (Raw) [21,22]. Briefly, anesthetized mice were tracheostomized and connected to a MiniVent ventilator (Hugo Sachs Elektronik, March, Germany), then ventilated with a tidal volume of 250 μl and a respiratory frequency of 120 breaths/minute. The mice were placed inside whole-body plethysmographs (Buxco) to measure Raw. Increasing doses of Mch were administered by ultraneblization for 3 minutes. The concentration of Mch that induced a 100% increase in Penh or Raw was expressed as PC_200_Mch (μg/ml) or PC_200_Mch Raw (μg/ml) as an indicator of AHR.

### Bronchoalveolar lavage (BAL) and histological examination

Bronchoalveolar lavage fluid (BALF) analyses were performed as described previously [19,20]. The lungs were lavaged four times with SA (0.5 ml each), and approximately 1.6 ml was consistently recovered with gentle handling. The cell suspension was centrifuged at 1,500 rpm for 10 minutes at 4°C. The cells were resuspended into 1 ml of saline with 1% BSA, and the total cell numbers were counted with a hemocytometer. Cytospin samples were prepared by centrifuging the suspensions (200 μl) at 300 rpm for 10 minutes. To clearly distinguish eosinophils from the neutrophils, three different stains were applied: Diff-Quick stain, May-Grünwald-Giemsa stain, and Eosino (Hansel) stain [19]. On the basis of the findings with these stainings, cell differentials were counted with at least 300 leukocytes in each sample. Lung histological examinations were performed as described previously [19,20]. Serum immunoglobulin (Ig) E and BALF cytokine concentrations were measured using ELISA kits (Pharmingen, SanDiego, CA) according to the manufacturer's instructions. The lower limits of sensitivity for the ELISA were 78 ng/ml (IgE), 7.8 pg/ml (IL-4), 15.6 pg/ml (IL-5), and 31.2 pg/ml (interferon (IFN)-γ), respectively.

### Fluorescence study and immunohistochemistry

On day 18, spleen cells (5 × 10^7^) from OVA-sensitized mice were labeled with fluorescent dye (PKH67; Sigma), and then transferred into syngenic recipients. In another experiment, CD4^+^CD62L^low ^cells (4 × 10^6^) from OVA-sensitized mice were positively selected as described in "depletion and positive selection study", and then labeled and transferred. On day 19, after perfusion with saline, lungs were excised. Five-micrometer sections were cut and observed by fluorescence microscopy (BX51; Olympus, Melville, NY). Immunohistochemistry was performed using Vectastain ABC kits (Vector Laboratories, Burlingame, CA) as described previously [23]. T cells were detected by staining for CD3 (cytoplasm, blue). Proliferation was assessed by staining for proliferating cell nuclear antigen (PCNA; nucleus, brown). Double-staining analysis of a single section was performed. Briefly, the tissue was deparaffinized and rehydrated with decreasing concentrations of ethyl alcohol. The slides were boiled in 0.05 M citric acid for 7 minutes. After cooling down to room temperature, endogenous peroxidase activity was blocked by incubating the slides in 3% H_2_O_2 _in methanol for 60 minutes. Next the slides were treated with blocking solution containing 5% normal rabbit serum, 2% casein, and 3% BSA for 45 minutes. Anti-CD3 Ab (5 μg/ml; Santa Cruz Biotechnology, Santa Cruz, CA) was applied to the tissue and incubated at 37°C for 30 minutes. After washing with PBS, biotinylated rabbit anti-goat IgG Ab was applied and incubated at 37°C for 30 minutes. After washing, avidin-biotin alkaline phosphatase complex was applied and incubated at 37°C for 30 minutes, followed by the addition of substrate solution. Color development was stopped by rinsing the slides in distilled water. Then, the slides were treated with blocking solution containing 5% normal goat serum, 2% casein, and 3% BSA for 45 minutes. Anti-PCNA Ab (2 μg/ml; Santa Cruz Biotechnology) was applied to the tissue and incubated at 37°C for 30 minutes. After washing with PBS, biotinylated goat anti-mouse IgG Ab was applied and incubated at 37°C for 30 minutes. After washing, avidin-biotin peroxidase complex was applied and incubated at 37°C for 30 minutes, followed by the addition of diaminobenzidine solution. Color development was stopped by rinsing the slides in distilled water. The slides were counterstained with neutral red. Positively immunostained cells were enumerated directly in 20 random high power fields (hpf; 40× objective).

### Depletion and positive selection study

For depletion, on day 18, spleen cells from OVA-sensitized mice were incubated with biotinylated anti-CD4 monoclonal antibody (mAb; RM4-5; Pharmingen), anti-CD8 mAb (53-6.7; Pharmingen), anti-CD11b mAb (M1/70; Pharmingen), anti-CD11c mAb (HL3; Pharmingen), or anti-CD19 mAb (1D3; Pharmingen), and then incubated with streptavidin-microbeads (Miltenyi Biotech, Bergisch Gladbach, Germany). For depletion of invariant Vα14 (iVα14) natural killer T (NKT) cells, spleen cells from OVA-sensitized mice were incubated with α-galactosylceramide (α-GalCer; kindly provided by the Pharmaceutical Research Laboratory of Kirin Brewery Company, Gunma, Japan)-loaded mouse CD1d: Ig dimeric protein (Pharmingen) and then incubated with anti-mouse IgG1-microbeads (Miltenyi Biotech). Bead-bound cells were depleted using magnetic separation columns. Flow cytometry confirmed that greater than 98% of CD4^+^, CD8^+^, CD11b^+^, CD11c^+^, or CD19^+ ^cells were removed from splenocytes, and 88% of cells that bound α-GalCer-loaded mouse CD1d dimer were removed (data not shown). Syngenic recipients received CD4^+^, CD8^+^, CD11b^+^, CD11c^+^, CD19^+^, or iVα14 NKT cell-depleted splenocytes (5 × 10^7 ^each). For positive selection, on day 18, spleen cells from OVA-sensitized mice were incubated with anti-CD4 mAb-coated or anti-CD11c mAb-coated microbeads (Miltenyi Biotech). Bead-bound cells were then isolated using magnetic separation columns. The purities of the enriched CD4^+ ^and CD11c^+ ^cells were 95% and 85%, respectively (data not shown). Over 95% of the CD4^+ ^cells were CD3^+ ^T cells (data not shown). Syngenic recipients received CD4^+ ^(1.25 × 10^7^) or CD11c^+ ^(1 × 10^6^) cells. We prepared a CD4^+^CD62L^low ^subset or a CD4^+^CD62L^high ^subset using an anti-FITC multisort kit (Miltenyi Biotech), FITC anti-CD4 mAb (RM4-5; Pharmingen), and anti-CD62L mAb-coated microbeads (Miltenyi Biotech). The purities of each subset were 80% (data not shown). Syngenic recipients received CD4^+^CD62L^low ^(4 × 10^6^) or CD4^+^CD62L^high ^(8.5 × 10^6^) cells. AR was measured on day 22 (4 days after the transfer).

### *In vitro *OVA stimulation and polarization to Th1 or Th2 phenotype

On day 18, spleen cells (5 × 10^6 ^cells/ml) from OVA-sensitized mice were incubated with OVA (200 μg/ml) for 4 days *in vitro*. On day 22, syngenic recipients received these stimulated cells (1 × 10^6^). In some experiments, dead cells were removed from cultured splenocytes using Percoll (Pharmacia Biotech, Uppsala, Sweden) gradient centrifugation. For polarization to Th1 cells, recombinant IL-12 (10 ng/ml; Genzyme Techne, Minneapolis, MN) and anti-IL-4 Ab (0.1 μg/ml; Genzyme Techne) were added to the culture medium. For polarization to Th2 cells, recombinant IL-4 (100 ng/ml; Genzyme Techne) and anti-IL-12 Ab (0.25 μg/ml; Genzyme Techne) were added. Polarization was confirmed by measuring IL-4 and IFN-γ in supernatant using ELISA. On day 22, CD4^+ ^T cells were positively selected, and syngenic recipients received CD4^+ ^Th1 cells or CD4^+ ^Th2 cells (5 × 10^5 ^each). AR was measured on day 26 (4 days after the transfer).

### *In vitro *proliferation and cytokine assays

Positively selected CD4^+ ^T cells, CD4^+^CD62L^high ^T cells, and CD4^+^CD62L^low ^T cells (2.5 × 10^5 ^cells/well, respectively) from OVA-sensitized mice were cultured with freshly isolated mitomycin C (Sigma)-treated splenocytes (2.5 × 10^5 ^cells/well) in the presence or absence of OVA. After 48 hours, the proliferation was assessed by a cell proliferation ELISA bromodeoxyuridine (BrdU) kit (Roche Applied Science, Mannheim, Germany). After 72 hours, cytokine concentrations in the supernatants were measured using ELISA.

### Statistics

Values are expressed as means ± SEM. Statistical analysis was performed by one-way ANOVA followed by Fisher's least significant difference test or Student's *t *test. A p value < 0.05 was considered significant.

## Results

### Passive cell transfer of spleen cells from antigen-sensitized mice induces AHR

**Figure 1:**
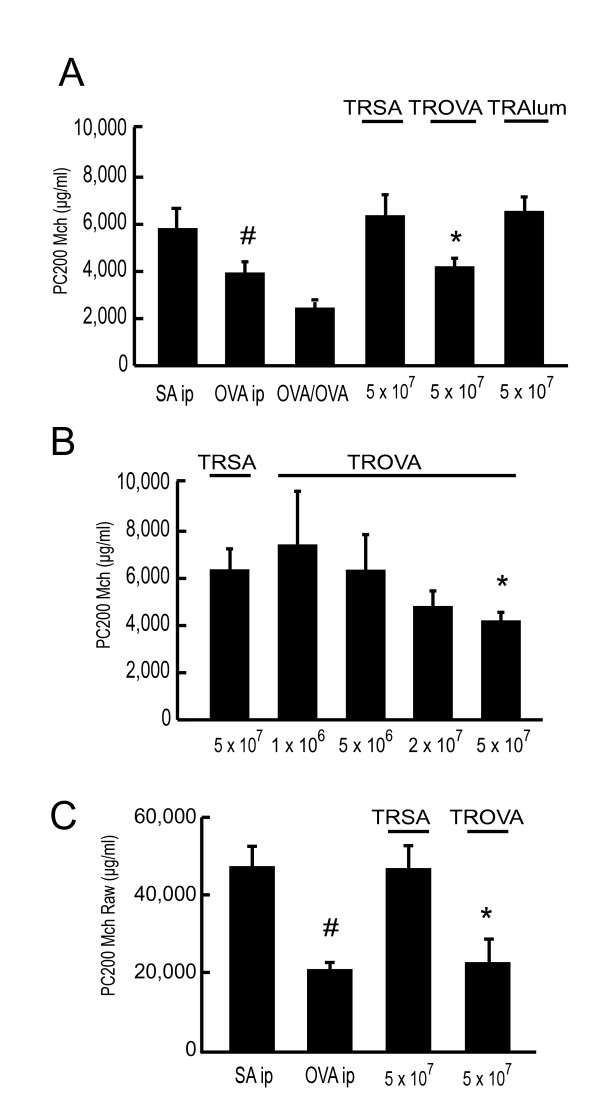
**Passive cell transfer of spleen cells from OVA-sensitized mice induces airway hyperresponsiveness (AHR)**. (A) Transfer of spleen cells from OVA-sensitized mice induces a moderate AHR. Mice were sensitized with OVA or SA on days 0 and 11. On day 18, recipients received the spleen cells (5 × 10^7^) from SA (without alum)-treated mice (TRSA), OVA-sensitized mice (TROVA), or SA (with alum)-treated mice (TRAlum). Airway responsiveness (AR) to methacholine (Mch) was measured with Penh methods on day 22 (4 days after the transfer) as described in Methods. Some OVA-sensitized mice were inhaled with OVA from day 18 to day 20. AR was measured on day 18 in mice received that i.p SA injection (SAip) or OVA injection only (OVAip), or on day 21 in mice that received OVA-sensitization and -inhalation (OVA/OVA). Values are presented as means ± SEM for 5 to 14 mice per group. * p < 0.05 compared with PC_200_Mch of TRSA (5 × 10^7^). # p < 0.05 compared with PC_200_Mch of SAip. (B) AR to Mch increases in a transferred-cell-number-dependent manner (n = 5–14 per group). Recipients received spleen cells from SA-treated mice (TRSA; 5 × 10^7^) or OVA-sensitized mice (TROVA; 1 × 10^6^, 5 × 10^6^, 2 × 10^7^, or 5 × 10^7^). AR was measured 4 days after the transfer. * p < 0.05 compared with PC_200_Mch of TRSA (5 × 10^7^). (C) AR was assessed by measurement of airway resistance (Raw) (n = 6–12 per group). * p < 0.05 compared with PC_200_Mch Raw of TRSA (5 × 10^7^). # p < 0.05 compared with PC_200_Mch Raw of SAip.

**Figure 2:**
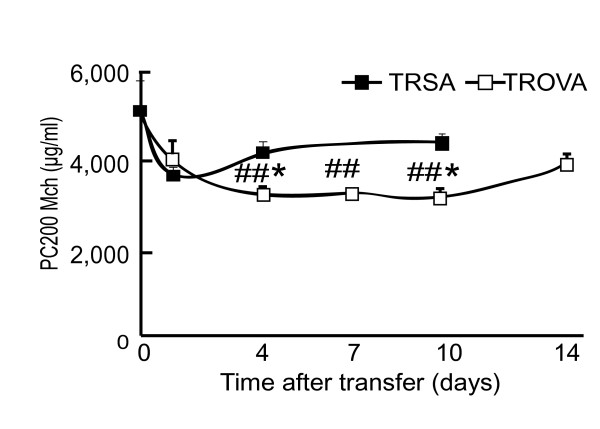
**Change in AHR following cell transfer**. Recipients received spleen cells (5 × 10^7^) from SA-treated mice (TRSA) or OVA-sensitized mice (TROVA). AR was measured at the indicated time after the transfer (n = 8 per group). ## p < 0.01 compared with the baseline value (before the transfer) (ANOVA). * p < 0.05 compared with PC_200_Mch at the same time point of TRSA.

As reported previously [19], immunization with OVA alone induced a significant increase in AR (OVA ip; PC_200_Mch; 3,870 ± 518 μg/ml) as compared with saline injection (SAip; 5,725 ± 1,009 μg/ml; Figure 1A). Injection with alum alone provoked a slight non-specific increase in AR, but it was not significant (data not shown). When OVA-sensitized mice received OVA inhalation challenges, then prominent infiltration of eosinophils was provoked and AR further increased (OVA/OVA; 2,564 ± 343 μg/ml; Figure 1A). In the group of mice that received spleen cells from OVA-sensitized mice (defined as "TROVA-mice"; 5 × 10^7^), PC_200_Mch was 4,191 ± 203 μg/ml, which was significantly lower than that of the group of mice that received the same number of spleen cells from SA-treated mice (defined as "TRSA-mice"; 5 × 10^7^; 6,357 ± 835 μg/ml; Figure 1A). Transfer of spleen cells from mice that were injected with SA plus alum did not induce AHR (TRAlum; 6,596 ± 697 μg/ml). Therefore, transfer of OVA-sensitized spleen cells reconstituted moderate AHR in a naive mouse to a similar degree of AHR induced by systemic sensitization with OVA antigen. In the TROVA-mice, AR increased in a cell-number dependent-manner (Figure 1B; 7,415 ± 2,176 μg/ml (1 × 10^6^), 6,343 ± 1,392 μg/ml (5 × 10^6^), 4,803 ± 572 μg/ml (2 × 10^7^), respectively). To confirm the reliability of the data obtained from the Penh system, we examined AR by measuring Raw with ventilated mice treated with the same immunization protocol. Similar results on AHR were obtained by measuring Raw (Figure 1C; PC_200_Mch Raw; SAip, 47,205 ± 4,767 μg/ml, OVA ip, 20,668 ± 1,562 μg/ml, TRSA, 46,702 ± 6,653 μg/ml, TROVA, 22,450 ± 9,535 μg/ml). So we used Penh system for the following experiments. In a time course study, the TROVA-mice revealed a significant increase in AR from 4 to 10 days after the transfer (Figure 2).

### Antigens that elicit Th2-type, but not Th1-type, immune response can induce AHR by cell transfer

**Figure 3:**
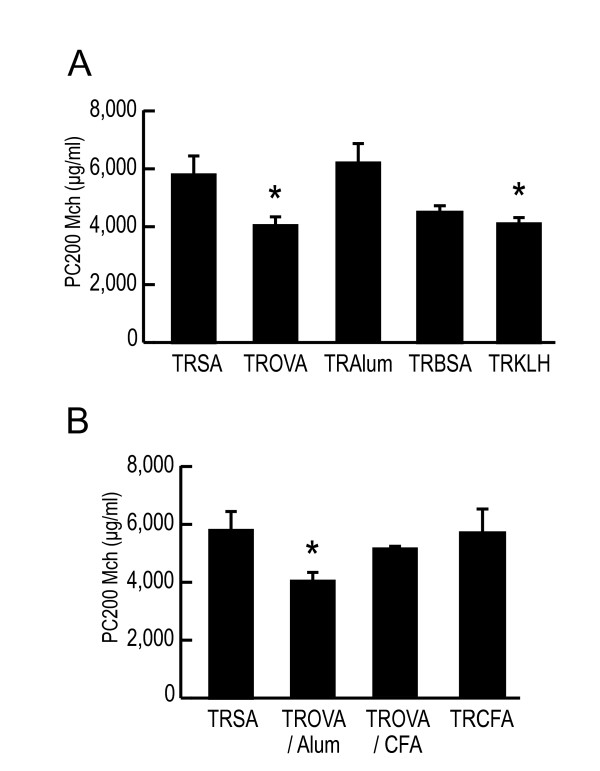
**Antigens that elicit Th2 type response, but not Th1 type response, induce transfer-mediated AHR**. (A) Antigens that elicit Th2 type response induce transfer-mediated AHR. Recipients received spleen cells (5 × 10^7^) from SA (without alum)-treated mice (TRSA), from OVA-sensitized mice (TROVA), from SA (with alum)-treated mice (TRAlum), from BSA-sensitized mice (TRBSA), or from KLH-sensitized mice (TRKLH). AR was measured 4 days after the transfer (n = 4–9 per group). * p < 0.05 compared with PC_200_Mch of TRSA. (B) CFA, an adjuvant that elicits Th1 type response, does not induce transfer-mediated AHR. Recipients received spleen cells (5 × 10^7^) from SA-treated mice (TRSA), from OVA (with alum)-sensitized mice (TROVA/Alum), from OVA (with CFA)-sensitized mice (TROVA/CFA), or from CFA-treated mice (TRCFA). AR was measured 4 days after the transfer (n = 4–9 per group). * p < 0.05 compared with PC_200_Mch of TRSA.

Next we confirmed other antigens than OVA could also induce AHR. Transfer of spleen cells from BSA-sensitized mice or KLH-sensitized mice also induced a significant AHR (Figure 3A; TRSA, 5,814 ± 638 μg/ml, TROVA, 4,112 ± 147 μg/ml, TRAlum, 6,224 ± 680 μg/ml, TRBSA, 4,633 ± 279 μg/ml, TRKLH, 4,123 ± 280 μg/ml). In another experiment, we confirmed systemic sensitization alone with BSA or KLH induced AHR without eosinophilic inflammation (data not shown). We also confirmed that systemic sensitization with BSA or KLH increased serum IgE concentration (data not shown). When BSA or KLH sensitized mice received airway antigen challenge, eosinophilic airway inflammation was provoked (data not shown). On the other hand, use of CFA instead of alum as an adjuvant, which is known to elicit Th1-type immunity [24], did not induce transfer-mediated AHR (Figure 3B; TRSA, 5,814 ± 638 μg/ml, TROVA/Alum, 4,112 ± 147 μg/ml, TROVA/CFA, 5,224 ± 74 μg/ml, TRCFA, 5,708 ± 945 μg/ml). Therefore, it could be generally considered that antigens that elicit Th2-type immune response could induce a significant AHR by cell transfer.

### The transfer-mediated AHR is provoked in an eosinophil-independent manner

**Figure 4:**
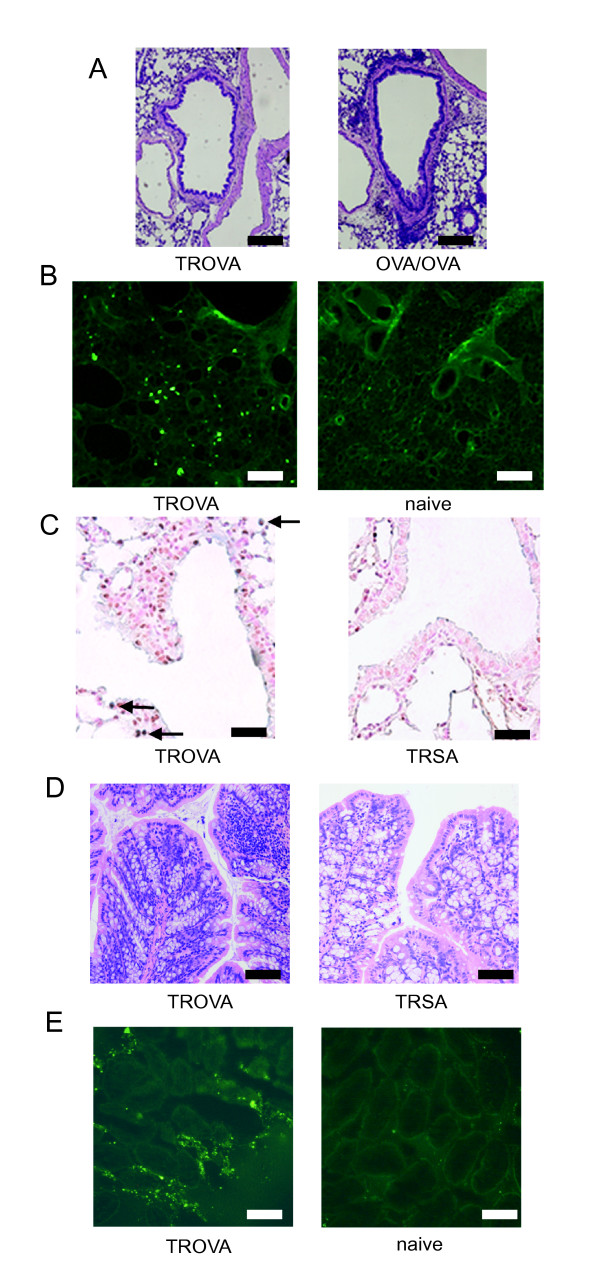
**Histologic findings**. (A) H&E stain. Lung sections from mice that received spleen cells from OVA-sensitized mice (TROVA) and from mice that received OVA-sensitization and aerosol OVA-challenge (OVA/OVA) are shown. Scale bar, 200 μm. (B) Fluorescence study. Spleen cells from OVA-sensitized mice were labeled with fluorescent dye (PKH67), and then transferred into recipients. Five-micrometer sections of the lungs were observed by fluorescence microscopy 24 hours after the transfer (TROVA). A lung section from naive mice without transfer is shown (naive). Scale bar, 100 μm. (C) Double staining analysis of a single section by immunohistochemistry. T cells were detected by staining for CD3 (cytoplasm, blue). Proliferation was assessed by staining for PCNA (nucleus, brown). Lung sections from mice that received spleen cells from OVA-sensitized mice (TROVA) and from mice that received spleen cells from SA-treated mice (TRSA) are shown. Proliferating T cells were clearly detected (black arrow). Scale bar, 40 μm. (D) Histologic findings of colon (H&E stain). Colon sections from mice that received spleen cells from OVA-sensitized mice (TROVA) and from SA-treated mice (TRSA) are shown. Scale bar, 200 μm. (E)Transferred cells recruit into the colon. Spleen cells from OVA-sensitized mice were labeled, and transferred. Five-micrometer sections of the colons were observed by fluorescence microscopy 24 hours after the transfer (TROVA). A colon section from naive mice without transfer is shown (naive). Scale bar, 100 μm.

In the TROVA-mice, the number of total cell, macrophage, and lymphocyte in BALF slightly increased, whereas eosinophil number did not (Table 1). In the TROVA-mice, a slight infiltration of inflammatory cells into the peribronchial area was detected in some specimens. However, prominent infiltration of eosinophils was not detected (Figure 4A, left). In contrast, in the OVA-sensitized and challenged mice (OVA/OVA), prominent infiltration of eosinophils into the peribronchial interstitial area or bronchial wall was observed (Figure 4A, right). These results indicated that cell transfer induced AHR without prominent infiltration of eosinophils in the lung.

**Table 1:** BALF findings

	Total cells(×10^2^)	Macrophage(×10^2^)	Lymphocyte(×10^2^)	Neutrophil(×10^2^)	Eosinophil(×10^2^)
TRSA 5 × 10^6^	919 ± 115	893 ± 111	25 ± 6	1 ± 1	0 ± 0
TROVA 1 × 10^6^	980 ± 149	945 ± 140	33 ± 7	3 ± 3	0 ± 0
TROVA 5 × 10^6^	1070 ± 132	1030 ± 132	34 ± 4	6 ± 4	0 ± 0
TROVA 2 × 10^7^	950 ± 78	898 ± 77	44 ± 7	6 ± 2	3 ± 1
TROVA 5 × 10^7^	1004 ± 105	942 ± 98	55 ± 11	6 ± 2	1 ± 1

Values are presented as means ± SEM for 5 to 14 mice per group.

### Some transferred cells recruit into the lung, and some T cells proliferate without further airway antigen challenge

We next analyzed lung sections from mice that received fluorescently labeled spleen cells from OVA-sensitized mice. The transferred cells were clearly detected in the lung 24 hours after the transfer, particularly in lung interstitial areas (Figure 4B, left). In contrast, the mice that had not received cell transfer did not show this finding (Figure 4B, right). Similar results were observed 3 days after the transfer (data not shown). Immunohistochemistry revealed that some T cells in the lung proliferated without further airway antigen challenge in the TROVA-mice (1.6 ± 0.2/hpf; Figure 4C, left). In contrast, in the TRSA-mice, proliferation of T cells was less detected (0.5 ± 0.1/hpf; Figure 4C, right).

### Transferred cells also distribute in other tissues and induce mild inflammation

In the TROVA-mice, a slight infiltration of inflammatory cells was also detected in the mucosa of colon in some specimens (Figure 4D, left). In contrast, it was less detected in the TRSA-mice (Figure 4D, right). Similar results were obtained in tissues other than colon such as stomach and small intestine (data not shown). Fluorescence study revealed that transferred cells also distributed among other tissues such as colon (Figure 4E), stomach, liver, and spleen (data not shown). Therefore, transferred cells did not recruit specifically into the lung, but distributed throughout the body.

### Th2 cell-type cytokines, but not IgE, mediate transfer-induced AHR

**Figure 5:**
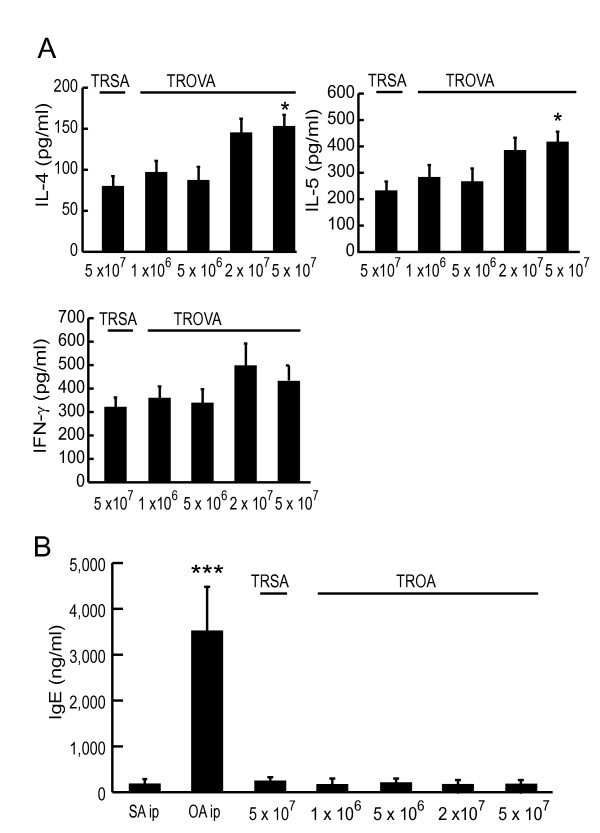
**Concentrations of BALF cytokines and serum IgE**. (A) BALF cytokine concentrations. Four days after the transfer, BAL was performed and then the centrifuged supernatant was assayed for IL-4, IL-5, and IFN-γ concentrations by ELISA, respectively (n = 5–14 per group). * p < 0.05 compared with the values of TRSA (5 × 10^7^). (B) Serum IgE concentrations (n = 5–14 per group). *** p < 0.001 compared with the values of SAip.

In the TROVA-mice (5 × 10^7^), the concentrations of IL-4 and IL-5 were significantly higher than those of the TRSA-mice (5 × 10^7^) (Figure 5A). The concentrations of IL-13 (data not shown) and IFN-γ levels (Figure 5A) also slightly increased in the TROVA-mice (5 × 10^7^), but these values were not significantly different from those of the TRSA-mice (5 × 10^7^). Thus, Th2 cell-type cytokines increased in BALF, and their increases might play a pivotal role in the transfer-mediated AHR. We also measured serum IgE concentration. No significant increase in IgE was detected (Figure 5B), suggesting that IgE did not mediate transfer-induced AHR.

### IL-4 plays an important role in transfer-mediated AHR

**Figure 6:**
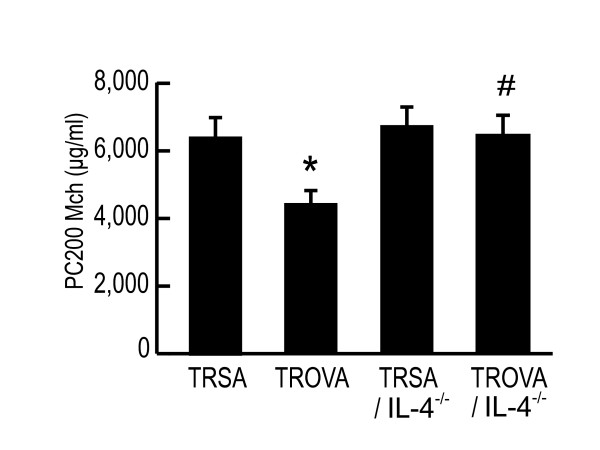
**IL-4 plays an important role in transfer-mediated AHR**. Recipients received spleen cells (5 × 10^7^) from SA-treated wild-type mice (TRSA), from OVA-sensitized wild-type mice (TROVA), from SA-treated IL-4-deficient mice (TRSA/IL-4^-/-^), or from OVA-sensitized IL-4-deficient mice (TROVA/IL-4^-/-^). AR was measured 4 days after the transfer (n = 6–8 per group). * p < 0.05 compared with PC_200_Mch of TRSA. # p < 0.05 compared with PC_200_Mch of TROVA.

As reported previously [19], IL-4 played a pivotal role in AHR that induced by antigen sensitization alone. So, we next examined the role of IL-4 in this transfer-mediated AHR. Transfer of spleen cells from OVA-sensitized, IL-4-deficient mice failed to induce the development of AHR (Figure 6; TRSA, 6,554 ± 758 μg/ml, TROVA, 4,209 ± 287 μg/ml, TRSA/IL-4^-/-^, 6,723 ± 765 μg/ml, TROVA/IL-4^-/-^, 6,593 ± 698 μg/ml) and an increase in BALF IL-4 concentration was not detected (data not shown). These results suggested that IL-4 production by OVA-sensitized spleen cells played an important role in the induction of transfer-mediated AHR.

### *In vitro *OVA stimulation potentiates the intensity of transfer-mediated AHR

**Figure 7:**
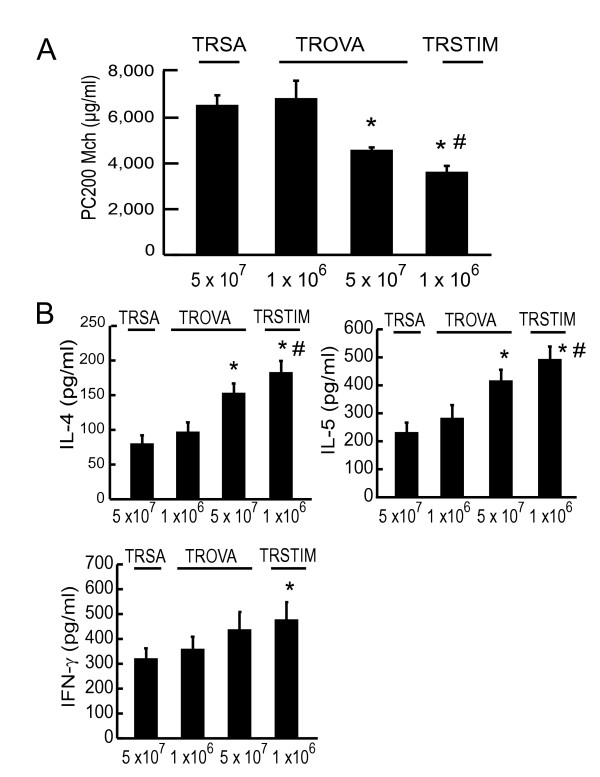
***In vitro *OVA stimulation increases the intensity of transfer-mediated AHR**. (A) Effect of *in vitro *OVA stimulation on transfer-mediated AHR. On day 18, spleen cells (5 × 10^6 ^cells/ml) from OVA-sensitized mice were incubated with OVA (200 μg/ml) for 4 days *in vitro*. On day 22, recipients received these stimulated cells by intravenous injection (TRSTIM: 1 × 10^6^). AR was measured 4 days after the transfer (n = 5–10 per group). * p < 0.05 compared with PC_200_Mch of TRSA (5 × 10^7^). # p < 0.05 compared with PC_200_Mch of TROVA (1 × 10^6^). (B) BALF cytokine concentrations. Four days after the transfer, BAL was performed and then the centrifuged supernatant was assayed for IL-4, IL-5, and IFN-γ concentrations using ELISA, respectively (n = 5–10 per group). * p < 0.05 compared with the values of TRSA (5 × 10^7^). # p < 0.05 compared with the values of TROVA (1 × 10^6^).

In another experiment, OVA-sensitized spleen cells were stimulated with OVA *in vitro *and then transferred. This treatment increased the intensity of transfer-mediated AHR (Figure 7A; TRSA, 6,556 ± 703 μg/ml, TROVA (1 × 10^6^), 6,848 ± 997 μg/ml, TROVA (5 × 10^7^), 4,607 ± 205 μg/ml, stimulated cell-transferred mice (TRSTIM) (1 × 10^6^), 3,654 ± 459 μg/ml). BALF cytokine concentrations of the recipients were also increased by this treatment (Figure 7B). This result indicated that stronger antigen stimulus induced stronger immune response, which resulted in the induction of higher AHR.

### CD4^+ ^Th2 cells induce AHR

**Figure 8:**
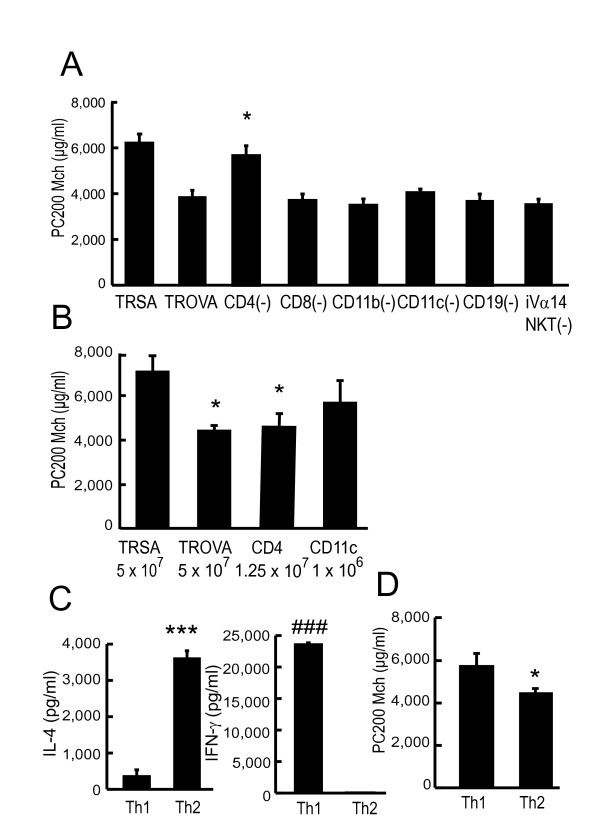
**CD4^+ ^Th2 cells directly induce AHR**. (A) Depletion study. Recipients received spleen cells (5 × 10^7^) from SA-treated mice (TRSA) or OVA-sensitized mice (TROVA). Other recipients received CD4^+^cell-depleted (CD4(-)), CD8^+ ^cell-depleted (CD8(-)), CD11b^+ ^cell-depleted (CD11b(-)), CD11c^+ ^cell-depleted (CD11c(-)), CD19^+ ^cell-depleted (CD19(-)), or iVα14 NKT cell-depleted (iVα14 NKT(-)) spleen cells from OVA-sensitized mice (5 × 10^7 ^each), respectively. AR was measured 4 days after the transfer (n = 6–10 per group). * p < 0.05 compared with PC_200_Mch of TROVA. (B) Effect of CD4^+ ^or CD11c^+ ^cells on transfer-mediated AHR. Recipients received unfractionated spleen cells from SA-treated mice (TRSA; 5 × 10^7^), or unfractionated (TROVA; 5 × 10^7^), CD4^+ ^(CD4; 1.25 × 10^7^), or CD11c^+ ^(CD11c; 1 × 10^6^) spleen cells from OVA-sensitized mice. AR was measured 4 days after the transfer (n = 5–8 per group). * p < 0.05 compared with PC_200_Mch of TRSA. (C) Polarization to Th1 or Th2 phenotype. On day 18, spleen cells (5 × 10^6 ^cells/ml) from OVA-sensitized mice were incubated with OVA (200 μg/ml) for 4 days *in vitro*. For polarization toward Th1 cells, IL-12 and anti-IL-4 Ab were added to the culture medium. For polarization toward Th2 cells, IL-4 and anti-IL-12 Ab were added. On day 22, positively selected CD4^+ ^T cells were cultured with freshly isolated mitomycin C-treated splenocytes and OVA (200 μg/ml). After 96 hours, IL-4 and IFN-γ concentrations in the supernatants were assayed. *** p < 0.001 compared with the value of the CD4^+ ^Th1 cells. ### p < 0.001 compared with the value of the CD4^+ ^Th2 cells. (D) Effect of Th1 or Th2 phenotype on transfer-mediated AHR. Recipients received CD4^+ ^Th1 or Th2 cells (5 × 10^5^). AR was measured 4 days after the transfer (n = 5–6 per group). * p < 0.05 compared with PC_200_Mch of mice that received CD4^+ ^Th1 cells.

To determine which cells are most important for this AHR induction, we carried out a cell depletion study. Transfer of CD4^+ ^cell-depleted splenocytes into naive mice failed to induce the development of AHR (Figure 8A; TRSA, 6,240 ± 577 μg/ml, TROVA, 3,858 ± 325 μg/ml, CD4 (-), 5,695 ± 543 μg/ml, CD8 (-), 3,738 ± 368 μg/ml, CD11b (-), 3,528 ± 327 μg/ml, CD11c (-), 4,077 ± 206 μg/ml, CD19 (-), 3,694 ± 434 μg/ml, iVα14 NKT (-), 3,497 ± 345 μg/ml) or failed to increase BALF cytokine concentrations (data not shown). We next carried out positive selection. We determined the numbers of CD4^+ ^and CD11c^+ ^spleen cells to be transferred based on the physiologic ratio of these cells (CD4^+ ^spleen cells were 25% and CD11c^+ ^spleen cells were 2% of total spleen cells, respectively). Transfer of positively selected CD4^+ ^T cells into naive mice induced AHR (Figure 8B; TRSA, 7,061 ± 831 μg/ml, TROVA, 4,381 ± 102 μg/ml, CD4, 4,526 ± 560 μg/ml, CD11c, 5,637 ± 1,040 μg/ml) and an elevation of BALF cytokine levels (data not shown), which were consistent with the results of depletion study. Next, we elucidated which of the two CD4-mediated response play a major role for AHR induction. Spleen cells were polarized to either Th1 or Th2 phenotype (Figure 8C) and each CD4^+^population was transferred. Transfer of CD4^+ ^Th2, but not Th1, cells induced AHR (Figure 8D; Th1, 5,732 ± 508 μg/ml, Th2, 4,384 ± 151 μg/ml).

### CD4^+^CD62L^low ^memory/effector T cells play an essential role in the transfer-induced AHR

**Figure 9:**
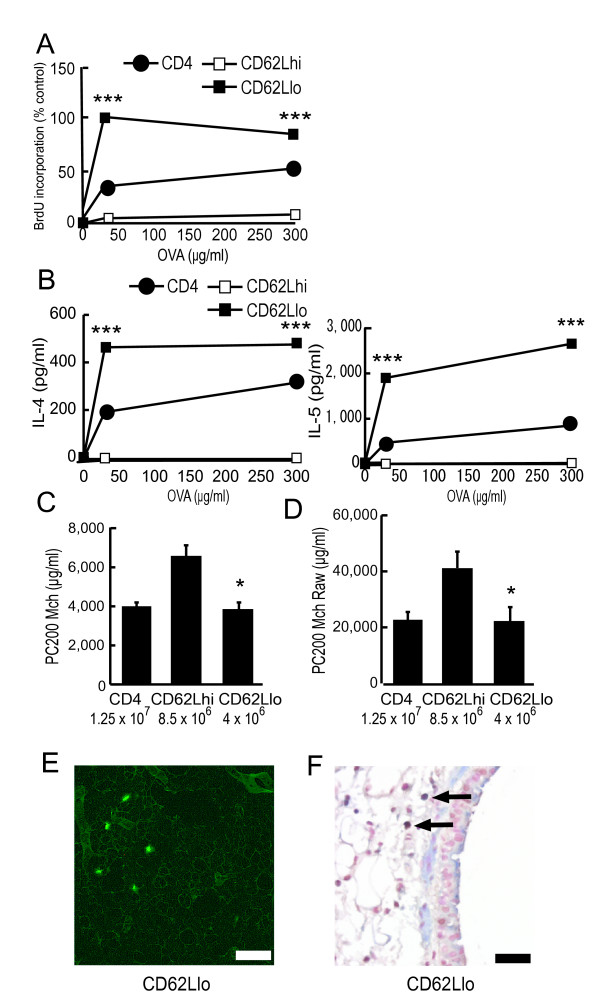
**CD4^+^CD62L^low ^memory/effector subset produces Th2-type cytokines and directly induces transfer-mediated AHR**. (A and B)CD4^+^CD62L^low ^memory/effector subset proliferates and produces Th2-type cytokines with antigen stimulation. On day 18, CD4^+ ^T cells (CD4), CD4^+^CD62L^high ^T cells (CD62Lhi), or CD4^+^CD62L^low ^T cells (CD62Llo) from OVA-sensitized mice were positively selected by magnetic cell sorting as described in Methods. Then, these cells were cultured with freshly isolated mitomycin C-treated splenocytes in the presence of OVA. After 48 hours, the proliferation was assessed by BrdU incorporation using ELISA (A). The maximum proliferation observed in response to OVA for CD4^+^CD62L^low ^T cells from OVA-sensitized mice was set as control (100%). After 72 hours, Th2-type cytokine levels in the supernatants were assayed using ELISA (B). *** p < 0.001 compared with the value of CD4^+^CD62L^high ^T cells. (C and D) CD4^+^CD62L^low ^memory/effector T cells induce AHR. Recipients received CD4^+ ^(CD4; 1.25 × 10^7^), CD4^+^CD62L^high ^(CD62Lhi; 8.5 × 10^6^), or CD4^+^CD62L^low ^(CD62Llo; 4 × 10^6^) cells from OVA-sensitized mice. AR was measured 4 days after the transfer. (C) AR was measured by Penh methods (n = 5–6 per group). * p < 0.05 compared with PC_200_Mch of mice that received CD4^+^CD62L^high ^T cells. (D) AR was assessed by measurement of Raw (n = 6–8 per group). * p < 0.05 compared with PC_200_Mch Raw of mice that received CD4^+^CD62L^high ^T cells. (E)Fluorescence study. CD4^+^CD62L^low ^cells from OVA-sensitized mice (4 × 10^6^) were labeled with fluorescent dye, and then transferred into recipients. Five-micrometer sections of the lungs were observed by fluorescence microscopy 24 hours after the transfer (CD62Llo). Scale bar, 100 μm. (E) Double staining analysis by immunohistochemistry. T cells were detected by staining for CD3 (cytoplasm, blue). Proliferation was assessed by staining for PCNA (nucleus, brown). Lung sections from mice that received OVA-sensitized CD4^+^CD62L^low ^cells (CD62Llo; 4 × 10^6^) are shown. Proliferating T cells were clearly detected (black arrow). Scale bar, 40 μm.

The results obtained so far indicated that antigen-stimulated CD4^+ ^Th2 cells reached the lung and proliferated there, then produced Th2-type cytokine, which resulted in the direct induction of AHR. They also indicated that antigen-pulsed memory/effector T cell phenotype might play an important role in the transfer-mediated AHR induction. So, finally we examined their role in our system. CD4^+^CD62L^high ^T cells and CD4^+^CD62L^low ^T cells were prepared by Ab-coated microbeads and column separation. In an *in vitro *study, the CD62L^low ^memory/effector subset proliferated and produced Th2-type cytokines in response to OVA, whereas the CD62L^high ^subset did not (Figure 9A and 9B). Moreover, the CD62L^low ^memory/effector subset from OVA-sensitized mice produced Th2-type cytokines even without further antigen stimulation, although the values were low (data not shown). Then, we performed transfer study. We determined the numbers of CD62L^high ^and CD62L^low ^cells to be transferred based on the physiologic ratio of these phenotypes (CD62L^high ^cells were 68% and CD62L^low ^cells were 32% of splenic CD4^+ ^T cells, respectively). Transfer of the CD62L^low ^memory/effector subset, but not the CD62L^high ^subset, induced AHR (Figure 9C; CD4, 3,968 ± 258 μg/ml, CD62Lhi, 6,549 ± 645 μg/ml, CD62Llo, 3,824 ± 420 μg/ml). When we evaluated AHR by measuring Raw, similar results were obtained (Figure 9D; PC_200_Mch Raw; CD4, 23,840 ± 3,350 μg/ml, CD62Lhi, 41,146 ± 6,451 μg/ml, CD62Llo, 22,146 ± 6,150 μg/ml). We also confirmed that some transferred CD4^+^CD62L^low ^cells actually recruited into the lung (Figure 9E). Moreover, in the mice that received the CD62L^low ^subset, some T cells in the lung proliferated there without further antigen stimulation (Figure 9F). These results strongly indicated that CD4^+^CD62L^low ^memory/effector T cells were essential for this transfer-mediated, antigen-induced AHR.

## Discussion

In the current study, we demonstrated that transfer of antigen-induced cellular immune response into naive mice reconstituted AHR in an antigen free setting. We found that CD4^+^CD62L^low ^Th2 cells play an essential role in this process. Our results strongly suggest that in sensitized individuals, memory/effector T cells could reach the lung tissue and locally act on the airways, thus would directly induce and maintain basal AHR independently of eosinophils, although the intensity could be moderate.

AHR is one of the most characteristic features of asthma [1]. However, the precise mechanism for its induction has not been fully clarified. It is considered that eosinophilic airway inflammation is closely linked to the AHR induction [1,4,5]. However, a causal link between eosinophilic airway inflammation and AHR has not been established. On the other hand, CD4^+ ^T cells, especially CD4^+ ^Th2 cells, are also involved in the induction of AHR [11,13-18]. However, the significance of eosinophils or CD4^+ ^T cells on the AHR induction has been clarified only in the effector phase, under the condition of airway antigen challenge. Therefore, the role of these cells in the AHR induction has not been evaluated in an antigen free setting. We previously reported that systemic antigen sensitization alone directly induces AHR before development of eosinophilic airway inflammation [19]. In addition, in the current study, transfer of spleen cells obtained from antigen-sensitized mice induced a significant AHR in naive mice without airway eosinophilia (Figure 1, Figure 3, Figure 4A, and Table 1). These results indicate that antigen-sensitized spleen cells could directly induce AHR. In humans, we previously reported that some patients with atopic dermatitis who are highly sensitized to mite antigen have a moderate AHR regardless of the lack of any history of asthma [25]. This would support the speculation that sensitization to an antigen could directly induce AHR also in humans.

We measured AR mainly by Penh system throughout the current study. Penh system has been widely used for measurement of AR to Mch in BALB/c mice [19,20,26]. Measuring Penh is superior to measuring Raw of ventilated mice in terms of its conciseness and non-invasiveness. In addition, sampling bias caused by maneuver in Penh system seemed to be lower than that in invasive ventilator system. Based on these advantages of Penh system, we could measure AR of large numbers of mice simultaneously with a good reproducibility. However, the accuracy of Penh as an indicator of AR has been recently criticized because Penh does not correlate with Raw especially in C57BL/6 mice [27-29]. Measuring Penh is more frequently affected by the heat and humidification than measuring Raw [30,31]. Considering these experimental and theoretical problems, we also examined AR by measuring Raw with ventilated mice in the most important experiments (Figures 1C and 9D). We confirmed that similar results were obtained by the two systems. Therefore, we considered that the data obtained from the Penh system in the current study were, to a certain extent, reliable.

Next we studied the mechanism of the cell transfer-induced AHR. The fluorescence study and double staining demonstrated that these transferred cells reached the lung and some T cells actually proliferated in the lung without further airway antigen challenge (Figure 4B and 4C). Byersdorfer *et al*. reported that some transferred Th1 cells migrate in the airway before antigen challenge [32]. Julia *et al*. reported that antigen sensitization alone distributes antigen-specific T cells in the BALF and in the lung [33]. These reports support the present findings. Therefore, some antigen specific T cells could have reached the lung in an antigen free setting. On the other hand, transferred cells reached the tissues other than lung such as colon, which induced a slight inflammation there (Figure 4D and 4E). These results suggested that antigen sensitization or transfer of antigen-sensitized spleen cells would distribute antigen-specific T cells among tissues, and thus could induce some immure response there even without local antigen challenge.

The result of BALF cytokine concentrations (Figure 5A) and transfer of CD4^+ ^Th2 cells (Figure 8D) demonstrated that CD4^+ ^Th2 cells played an essential role in the induction of the AHR. In addition, results from positive and negative selection studies clarified that CD4^+^CD62L^low ^T cells were essential for transfer-induced AHR induction, while other phenotypes such as CD4^+^CD62L^high ^T cells or iVα14 NKT cells were not (Figures 8 and 9). There are two distinct populations of memory CD4^+ ^T cells, which are distinguished by the expression of CCR7 [34]. One is CCR7^high^ central memory T cells which home preferentially to lymph nodes, another is CCR7^low ^effector memory cells which traffic more efficiently to non-lymphoid tissues and acquire effector function more rapidly [34,35]. Central memory T cells express high levels of CD62L [34]. In contrast, effector memory T cells express CD62L to a lower and variable extent [34]. Therefore, CD62L^low ^memory T cells are thought to be a subset of effector memory T cells [34,36]. We demonstrated that the CD62L^low ^subset proliferated and produced Th2-type cytokines in response to OVA, while the CD62L^high ^subset did not (Figure 9A and 9B). Moreover, transferred CD62L^low ^subset recruited into the lung, and proliferated there without local antigen challenge (Figure 9E and 9F). Therefore, in our system, some antigen specific T cells could have acquired a memory/effector phenotype (effector memory T cells), reached the lung, proliferated and produced cytokines, then finally induced AHR in an antigen free setting. Furthermore, local antigen stimuli would further accelerate immune response and thus airway eosinophilia would be induced [37], which could further increase AHR (Figures 1A and 4A).

So far, whether immunocompetent cells would modify intrinsic AHR has remained unclear. De Sanctis *et al*. reported that T lymphocytes mediate intrinsic AHR [38]. However, Hadeiba *et al*. later reported that transfer of CD4^+ ^T cells that are not stimulated with antigen does not alter the intrinsic AHR [39]. The latter finding is strongly supported by the present findings that antigen-stimulated cells mediated AHR induction, while cells that had not encountered antigen did not.

*In vitro *OVA stimulation of OVA sensitized spleen cells enhanced transfer activity to induce AHR (Figure 7A) and cytokine concentration in BALF (Figure 7B). Wise *et al*. reported that *in vitro *OVA stimulation enhances transfer activity to provoke eosinophilic airway inflammation [37]. We needed larger numbers of OVA-sensitized spleen cells to induce AHR than other studies [17,18], because we did not stimulate cells with OVA *in vitro *before transfer in most part of this study. Spleen cells from OVA-sensitized mice produced greater amount of cytokines upon further stimulation with OVA *in vitro *(data not shown). Therefore, the more Th2-type cytokine was produced by transferred cells, the greater the intensity of AHR increased (Figure 7).

Among cytokines produced by CD4^+ ^Th2 cells, which is most important for developing of AHR has not been fully clarified. IL-13 is a candidate for developing AHR independently of eosinophilic inflammation [12]. On the other hand, some previous studies [10,40] including ours [19] showed that IL-4 also plays an important role. In the current study, IL-4 and IL-5 concentrations in BALF increased by cell transfer (Figure 5), while IL-13 did not increase significantly (data not shown). Moreover, transfer of spleen cells from antigen-sensitized IL-4-deficient mice did not induce AHR (Figure 6). So in the present experimental system, IL-4 would play a major role. Airway smooth muscle cells express IL-4Rα [41,42]. We speculate that one of the mechanisms of AHR induction by cell transfer could be a direct influence on smooth muscle cells [43]. The relationship between Th2 cytokine and smooth muscle cell should be further elucidated. NKT cells are another cell source of IL-4 [44-47] and IL-13 productions [44], and would influence the subsequent adaptive immune response and T cell polarization [45,47]. The development of AHR is abrogated in NKT-cell deficient mice [44,46]. However, in the current study, iVα14 NKT cells did not influence this transfer-mediated AHR (Figure 8A). This indicates that although NKT cells might play some important role in a certain phase of sensitization, once conventional T cells are activated and gain effector function, iVα14 NKT cells would have little contribution to the induction of AHR.

## Conclusion

In conclusion, the current study clarified the significance of antigen-specific memory/effector CD4^+ ^T cell-mediated Th2-type immune response as an essential factor to induce basal AHR in an antigen independent setting. It would also propose that suppression of antigen-specific immune response itself should be a critical target for controlling allergic asthma.

## Competing interests

The author(s) declare that they have no competing interests.

## Authors' contributions

KN and MD carried out animal experiments and preparation of the manuscript. KO, YT, AS and RT carried out animal experiments. YK and KN assisted with positive/negative cell selections. KY participated in the direction of the study. All authors read and approved the final manuscript.
